# Immune response to live-attenuated Japanese encephalitis vaccine (JE-CV) neutralizes Japanese encephalitis virus isolates from South-East Asia and India

**DOI:** 10.1186/1471-2334-14-156

**Published:** 2014-03-21

**Authors:** Matthew Bonaparte, Bashir Dweik, Emmanuel Feroldi, Claude Meric, Alain Bouckenooghe, Stephen Hildreth, Branda Hu, Sutee Yoksan, Mark Boaz

**Affiliations:** 1 Sanofi Pasteur Global Clinical Immunology Department, Swiftwater, USA; 2 Sanofi Pasteur Clinical Development Department, Marcy l’Etoile, France; 3 Sanofi Pasteur Clinical Development, Singapore, Singapore; 4 Center for Vaccine Development, Institute of Molecular Bioscience, Mahidol University, Bangkok, Thailand

**Keywords:** Japanese encephalitis vaccines, Humoral immune response, JEV, Wild-type strains

## Abstract

**Background:**

During clinical development of the licensed Japanese encephalitis chimeric virus vaccine (JE-CV), the neutralization capacity of vaccine-induced antibodies was assessed against the vaccine virus and against well characterized wild-type (wt) viruses isolated between 1949–1991. We assessed whether JE-CV-induced antibodies can also neutralize more recent wt Japanese encephalitis virus (JEV) isolates including a genotype 1 isolate.

**Methods:**

Sera from 12–18 month-old children who received a single dose of JE-CV in a phase III study in Thailand and the Philippines (ClinicalTrials.gov NCT00735644) were randomly selected and pooled according to neutralization titer against JE-CV into eight samples. Neutralization was assessed by plaque reduction neutralization tests (PRNT_50_) against three recent isolates from JEV genotypes 1 and 3 in addition to four JEV previously tested.

**Results:**

Neutralization titers against the three recent JEV strains were comparable to those observed previously against other strains and the vaccine virus. The observed differences between responses to genotype 1 and 3 viruses were within assay variability for the PRNT_50_.

**Conclusions:**

The results were consistent with previously generated data on the neutralization of wt JEV isolates, immune responses induced by JE-CV neutralize recently isolated virus from southeast (SE) Asia and India.

## Background

Japanese encephalitis (JE) is a mosquito-borne viral disease that is seasonally endemic in many countries in Southeast Asia, with three billion people living in endemic areas [1]. Although most infections are sub-clinical, JE infection can cause febrile illness associated with central nervous system inflammation [2].

JE is a vaccine-preventable disease and several vaccines are currently in use [1,3,4]. A live, attenuated, JE chimeric virus vaccine (JE-CV), developed by replacing the pre-membrane and envelope coding sequences from the yellow fever vaccine virus (strain 17D) genome with the corresponding sequences from the JE SA14–14–2 virus strain [5,6], was approved in 2010 by the Therapeutic Goods Administration (TGA) in Australia and by the Thai Food and Drug Administration, and is known as IMOJEV™. In clinical development JE-CV was shown to be safe and immunogenic in adults and children with 99% and more than 95% of recipients seroprotected one month after the vaccination respectively [7,8].

JE virus (JEV), a member of the genus Flavivirus, is considered to exist as a single serotype and four main genotypes were identified initially on the basis of sequence within the pre membrane (prM) gene region [9,10]. More recently, the sequence of the envelope (E) gene region was proposed and used to define JEV genotypes, and using this method a fifth genotype was identified [11]. The JEV neutralizing antibody response is considered to be the correlate of protection for JE vaccines, with a plaque reduction neutralization 50% (PRNT_50_) titer of 1:10 defining the seroprotective threshold [12-14]. However, PRNT_50_ assays are performed mostly using the vaccine homologous virus [15,16], with neutralization responses against wild-type JEV in general less well characterized, perhaps unsurprising given the difficulty in isolating JEV from clinical infections. It is noteworthy that all licensed JE vaccines are based on genotype 3 viruses. Assessing vaccine-induced neutralization responses against circulating heterologous strains of JEV, particularly against JEV genotype 1 which is now the dominant genotype across parts of Asia, [17-19] provides a deeper understanding of a vaccine’s immunogenicity.

We previously demonstrated the ability of serum from adults and children vaccinated with JE-CV to neutralize heterologous wt JEV from genotypes 1–4 isolated between 1949 and 1991 [5,8]. Two recent publications with other JE vaccines suggested that genotype 1 JEV are less well neutralized than genotype 3 JEV [20,21]. We therefore sought to further characterize the immunogenicity of JE-CV by assessing neutralization against recent JEV isolates, with emphasis on whether there are differences between genotypes 1 and 3.

## Methods

### Serum pools from immunized toddlers

Sera were obtained from 12–18 month old children at who had received a single dose of JE-CV in a phase III study in Thailand and the Philippines (ClinicalTrials.gov NCT00735644; [22]).

Positive and negative sample pools were created using sera collected from these children on D28 post-vaccination and pre-vaccination, respectively. The selection of individual serum samples for pooling was based on JEV neutralizing antibody titers determined in JE-CV PRNT_50_ assay previously as part of the study [22]. Five different titer categories were created for pooling: negative (titers <10, one pool), low (titers 40–80, two pools) medium (titers 160–320, two pools), high (titers 640–2560, two pools), and very high (titers >5120, one pool). The medium titer sera pools most closely represent the response observed in the original study population that had a geometric mean titer (GMT) 95% confidence interval (CI) of 168–271 [22]. The other titer pools were created to assess the relationship between the neutralization titer against wt JEV, with the titer against the vaccine strain. Each pool included 7–20 individual samples, all except two of which were from children who were serologically JE-naïve before vaccination (one subject in each of the low titer pools had a JE-CV PRNT_50_ titer of ≥10). All except 3 of the samples were also serologically dengue-naïve before vaccination (indicated by dengue PRNT titer ≥10 to at least one serotype; one subject in one low titer pool, one subject in one medium titer pool, and one subject in the very high pool).

The Institutional Review Board of the Department of Pediatrics at Phramongkutklao hospital, Bangkok, Thailand, approved the protocol. The child’s parent or guardian provided signed informed consent as part of the original study procedures.

### JEV Viruses

Three isolates were obtained from the World Health Organization (WHO) Flavivirus Diagnostics Reference Laboratory for Asia at the Center for Vaccine Development University of Mahidol, Thailand: JEV-SM1 a genotype 1 JEV isolated from a pig in Thailand, 2003, JEV-902/97 a genotype 3 virus from a clinical case in Vietnam, 1997 and JEV-057434 another genotype 3 from a clinical case in India, 2005.

Three well characterized reference viruses were used, including two classified as genotype 3: Nakayama and SA14-14-2, and one from genotype 1: TVP-8236. The JE-CV vaccine virus, which is derived from SA14-14-2, was also tested [23] (Table 1).

**Table 1:** JEV used in JEV PRNT50 testing

**JEV**	**Genotype**	**Country of origin**	**Year of origin**	**Virus origin**	**Passage history of virus**	**Accession number**
JEV-057434	3	India	2005	Human	2 passages in mosquito cell lines; 3 passages in Vero cells	[Genbank:EF623988.1]
JEV-SM1	1	Thailand	2003	Pig	2 passages in mosquito cell lines; 3 passages in Vero cells	[Genbank:DQ087971.1]
JEV-902/97	3	Vietnam	1997	Human	1 passage C6/36 cells; 1 passage in suckling mouse; 3 passages in LLC-MK2 cells	[Genbank:JQ390453.1]
TVP-8236	1	Korea	1991	*Culex tritaeniorhynchus*	6 passages in mosquito cell lines; 6 passages in Vero cells	Not available
SA14-14-2	3	China	1954	Derived from SA14 isolated in *Culex pipiens* mosquitoe	Passages in mouse brain, PHK cells, mouse spleen, mouse skin, hamster spleen, with multiple intermediate plaque purifications in CEF [24]; 3 passages in Vero cells	[Genbank:AF315119.1]
Nakayama	3	Japan	1935	Human	11 passages in suckling mouse; 4 passages in Vero cells	[Genbank:EF571853.1]
JE-CV	3	Chimeric Virus, YF 17D with Envelope sequence from SA-14-14-2 [23]			5 passages in Vero cells	Not applicable

### Neutralization tests

Three independent assay runs were performed for each sera pool per virus by a single technician using the method detailed below:

In brief, serial 10-fold dilutions of the serum pools were mixed with a constant challenge dose of each respective JEV (45–60 plaques per well) and inoculated in duplicate, onto wells of a 6-well plate of confluent LLC-MK2 cells. After adsorption, cell monolayers were overlaid with carboxymethylcellulose (CMC)/neutral red and incubated for several days. Plaques were then enumerated in each well. The neutralizing antibody titer was calculated and expressed as the reciprocal serum dilution (1/dil) reducing the mean plaque count by 50% as calculated by probit analysis.

### Statistical methods

GMTs were calculated for each sample against the JEV using the three independently generated results per sample per JEV. The calculation of GMT from the 3 independent sample runs was performed to minimize any impact of assay variation on interpretation of the JEV neutralization. Normal assay variation is considered to be approximately one 2-fold difference in titer.

The seven JEV were ranked for neutralization sensitivity within each sample pool using GMT.

## Results

### Geometric mean titers

**Figure 1:**
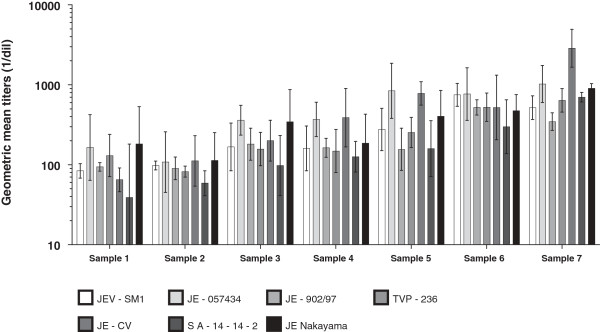
Geometric mean titers for the sera pools against the different JEV.

Each of the serum pools were tested in three independent assay runs in the respective wild type JEV and the GMT of the seven positive samples (+/- standard deviation) is presented in Figure 1. All seven strains were neutralized by pools of the JEV antibody-positive samples, whereas none were neutralized by the pooled JEV antibody-negative pre-vaccination samples (Table 2).

**Table 2:** GMTs of sera pools tested with different JEV

**Pool details**		**JEV PRNT**_**50 **_**neutralization results**							
**Sample no.**	**Pooled sera samples titer category**^**a**^	**JEV-SM1**^**b**^	**JE-057434**^**b**^	**JE-902/97**^**b**^	**TVP-8236**^**c**^	**JE-CV**^**c**^	**SA14-14-2**^**c**^	**JE Nakayama**^**c**^	**Sample overall GMT**
**1**	Low	**84** (68–103)	**165** (64–423)	**94** (83–106)	**130** (71–240)	**65** (46–91)	**39** (8–181)	**182** (62–533)	**96** (74–126)
**2**	Low	**98** (86–111)	**108** (45–258)	**90** (65–125)	**82** (70–96)	**112** (54–231)	**59** (41–84)	**113** (51-252)	**93** (81–106)
**3**	Medium	**167** (84–332)	**361** (235–554)	**181** (114–286)	**157** (97–254)	**200** (111–360)	**98** (41–232)	**344** (136–872)	**197** (158–246)
**4**	Medium	**160** (84–305)	**369** (225–604)	**163** (124–214)	**148** (80–276)	**388** (167–899)	**126** (81–196)	**186** (81–429)	**200** (161–249)
**5**	High	**276** (150–508)	**841** (380–1860)	**155** (85–286)	**253** (164–391)	**780** (557–1092)	**159** (71–355)	**404** (192–851)	**333** (244–456)
**6**	High	**749** (539–1040)	**766** (359–1634)	**522** (419–649)	**523** (348–788)	**520** (205–1319)	**299** (138–649)	**474** (298–753)	**529** (448–623)
**7**	Very high	**517** (368–728)	**1023** (600–1743)	**346** (269–446)	**638** (455–895)	**2865** (1660–4945)	**699** (609–803)	**902** (788–1034)	**801** (598–1074)
**8**^**d**^	Negative	<10	<10	<10	<10	<10	<10	<10	**<10**
**JEV Overall GMT**		**218** (152–313)	**397** (271–584)	**183** (138–244)	**214** (154–300)	**351** (201–613)	**138** (89–214)	**302** (218–418)	**243** (209–282)

GMTs are presented in bold font with 95% confidence intervals in parentheses.

^a^Results from original study testing using JE-CV PRNT assay at Focus Diagnostics Ltd., US [22]; low = titer range 40–80, medium = titer range 160–320, high = titer range 640–2560, very high = titer range >5120., negative = <10.

^**b**^Recent JEV isolate.

^**c**^JEV isolate tested in previous clinical trials [5,8].

^d^Sample 8 was not used in calculating overall (sample/JEV) GMT.

In each pool, GMTs against the seven strains were similar and generally within one 2-fold difference of each other (Figure 1 and Table 2). For example, the two low level JEV Ab positive samples showed titers between 39–182 and 59–113; the medium titer samples 98–361 and 126–388; high titer samples from 155–841 and 299–766; and very high titer pool from 346–2865 for the seven JEV strains, respectively. Overall, the results for each individual sample are within the expected assay variability for a functional assay, though the very high titer sample shows slightly higher variability.

The neutralization titers against the recent JEV were comparable to those against the vaccine virus and the non-contemporary wild-type JEV.

Genotype 1 and 3 strains were also neutralized with similar titers: titers of 160–167 against genotype 1 and 163–369 against genotype 3 were observed in the medium titer pool. GMTs against the vaccine virus ranged from 200–388, and against the non-contemporary wild-type strains ranged from 157–344 and 126–186 (Table 2).

GMTs against the vaccine virus was within the range of titers observed to the wild-type JEV in all sample pools, except in the very high titer pools where the GMT to vaccine virus was 3–8 fold higher.

### Relative neutralization sensitivities

Since the challenge dose of the virus used in the PRNT was similar for each virus (average input plaque count per well of 45–60), the relative neutralization sensitivity of the six different JEV and the vaccine virus was assessed. The sample neutralizing antibody GMTs were used to rank the JEV within each sample pool and to generate an average rank (Table 3). In general the differences in neutralization titers within samples to each JEV were not marked, and not statistically significant as the 95%CIs of the overall GMTs overlap. A trend for JE-057434, JE-CV, and JE Nakayama from genotype 3 as the most neutralization sensitive was observed, i.e., samples had higher titers against these JEV versus the other JEV tested. The two genotype 1 JEV were in the middle of this ranking, and SA14-14-2, an attenuated strain derived from a genotype 3 JEV and donor of the prM and E sequences of the JE-CV vaccine strain, was observed to be the least sensitive to neutralization.

**Table 3:** Ranking for JEV neutralization sensitivity within each sera pool

**Sample no.**	**JEV-SM1**	**JE-057434**	**JE-902/97**	**TVP-8236**	**JE-CV**	**SA14-14-2**	**JE Nakayama**
**1**	5	2	4	3	6	7	1^a^
**2**	4	3	5	6	2	7	1
**3**	5	1	4	6	3	7	2
**4**	5	2	4	6	1	7	3
**5**	4	1	7	5	2	6	3
**6**	2	1	4	3	5	7	6
**7**	6	2	7	5	1	4	3
**8**^**b**^	N/A	N/A	N/A	N/A	N/A	N/A	N/A
**Average rank**	**4.4**	**1.7**	**5.0**	**4.9**	**2.9**	**6.4**	**2.7**

Average rank is presented in bold font.

^a^Ranking performed from 1 being the most neutralization sensitive (i.e. the highest neutralization titers) to 7 being the lowest.

^**b**^N/A – Not applicable, sample 8 was negative for all virus strains and was not used in the ranking.

## Discussion

The assessment of vaccine immunogenicity is critical as a surrogate for assessment of clinical efficacy [13]. The immunogenicity of JE vaccines are usually assessed in terms of neutralizing antibodies against the vaccine virus or a homologous virus [15,16,22], however it is also important to characterize the response against heterologous circulating JEV. This study extends previous work, that documented the antibody responses to a reference panel of wild-type JEV [5,8], by showing that pooled sera from toddlers vaccinated with JE-CV also neutralize recently isolated wild-type virus, including genotype 1 virus [17-19]. Virus used in our studies were obtained from pig, mosquito and clinical infections in Vietnam, Thailand and India between 1997–2005. Other authors have assessed the ability of vaccine-induced antibodies to neutralize JEV obtained between 1935–1984 [23], with some more recent studies using JEV from 2002 [20] and 2009 [21].

Consistent with the classification of JEV viruses as a single serotype, we observed no marked differences in neutralization sensitivity between recent versus older isolates, and saw that the highest level of neutralization response was against the recent, genotype 3 isolate, JE-057434. We also saw no difference in the capacity to neutralize genotype 1 and genotype 3 viruses with titers generally within 2-fold of each other, confirming our previous observations both at 28 days and 6 months after vaccination [5,8]. It was observed that the neutralization response against the homologous vaccine strain, JE-CV, trended higher than the parental strain SA14-14-2. This may relate to the different cell lines used to passage the two viruses (Table 1). In another study, sera from individuals vaccinated with SA14-14-2 neutralized virus from genotype 1 with titers that were similar to or 1–4-fold lower than titers against genotype 3 viruses [25]. Our findings with serum collected after vaccination with JE-CV contrast with those in recent reports that used some of the same JEV strains [20,21]. With sera from subjects vaccinated with an inactivated Vero cell derived vaccine, there was a 10-fold difference in titers between assays performed with a genotype 1 virus, compared with the vaccine-homologous genotype 3 virus [20]. Similarly, in a separate study of a mouse brain derived vaccine titers were 3–8-fold lower to genotype 1 compared with the genotype 3 vaccine-homologous strain, and 2-3-fold lower than a heterologous genotype 3 strain [21].

It is unclear why differences in relative neutralization of genotype 1 JEV have been observed. JEV neutralization is not necessarily expected to correlate with genotype given that a single serotype of JEV exists, and that identification of genotypes 1 and 3 was based initially upon the prM gene [9,10], whereas neutralization is mediated primarily through the E protein [26]. However, some antigenic differences have been observed between genotype 1 and 3 strains using JEV Env specific mAb, although the majority of mAb tested bound to strains from both genotypes [27].

Another possibility may relate to methodological differences in either the laboratory culture of JEV or the serological assays or both, although this is not apparent in the case of one of these studies as the same assay and some of the same viruses were used [20]. Furthermore, in our data a relationship between the number of laboratory passages and neutralization sensitivity across these JEV is not apparent (data not shown).

One factor that does differ between these studies is the type of vaccine given: neutralizing responses induced by live attenuated vaccines (JE-CV and SA14-14-2) were not inferior against genotype 1 virus compared to homologous virus [5,8,25], while responses induced by inactivated vaccine were [20,21]. However, this contrasts somewhat to the first inactivated JEV vaccines that were shown to neutralize heterologous JEV isolates, and also provide protection against disease in Taiwan and Thailand reviewed in [28].

The similar levels of neutralization seen after vaccination with JE-CV in our study suggests that the recent emergence of genotype 1 JEV in replacement of genotype 3 [17,18,29,30], and the continued observation of isolates with varied sequences [31], are not necessarily a cause for concern. Epidemiological data do not indicate a noticeable increase in JE disease that presumably would be attributable to genotype 1 [17,18,29]. However given the conflicting neutralization findings in the literature, the epidemiology of JEV and any genotype associations should continue to be monitored [19,30].

## Conclusions

Immune responses induced in toddlers by vaccination with the licensed JE-CV vaccine were able to neutralize recent wild-type viruses circulating in SE Asia and India, with similar titers compared to the vaccine strain and other wild-type strains. This finding is reassuring given the constant evolution of the virus and of its geographic distribution in Asia.

## Abbreviations

CMC: Carboxymethylcellulose; E: Envelope; JE: Japanese encephalitis; JE-CV: JE chimeric virus; JEV: JE virus; prM: Pre membrane; PRNT50: 50% plaque reduction neutralization; SE: Southeast; TGA: Therapeutic goods administration; WHO: World Health Organization; wt: Wild-type; 1/dil: Reciprocal dilution.

## Competing interests

Emmanuel Feroldi, Mark Boaz, Matthew Bonaparte, Bashir Dweik, Stephen Hildreth, Branda Hu, and Alain Bouckenooghe are employees of Sanofi Pasteur; Sutee Yoksan has received research grants from Sanofi Pasteur.

## Authors’ contributions

CM conceived this experimental research study, MBZ, MB and BH determined the design and EF and AB ensured this study was feasible. SY carried out serology, BD conducted the statistical analysis. MBZ, MB, BH, SH, CM contributed to the data analysis and data interpretation. MBZ drafted the manuscript and all authors read and approved the final manuscript.

## Pre-publication history

The pre-publication history for this paper can be accessed here:

http://www.biomedcentral.com/1471-2334/14/156/prepub

## References

[B1] Halstead SB Jacobson J Plotkin SA, Orenstein WA, Offit PA Japanese encephalitis vaccines Vaccines 2008 5 Philadelphia (PA): Saunders Elsevier 311 352

[B2] Mackenzie JS Gubler DJ Petersen LR Emerging flaviviruses: the spread and resurgence of Japanese encephalitis, West Nile and dengue viruses Nat Med 2004 14 12 Suppl S98 S109 15577938 10.1038/nm1144

[B3] Beasley DW Lewthwaite P Solomon T Current use and development of vaccines for Japanese encephalitis Expert Opin Biol Ther 2008 14 1 95 106 10.1517/14712598.8.1.95 18081539

[B4] Halstead SB Thomas SJ New Japanese encephalitis vaccines: alternatives to production in mouse brain Expert Rev Vaccines 2011 14 3 355 364 10.1586/erv.11.7 21434803

[B5] Monath TP Guirakhoo F Nichols R Yoksan S Schrader R Murphy C Blum P Woodward S McCarthy K Mathis D Johnson C Bedford P Chimeric, live, attenuated vaccine against Japanese encephalitis (ChimeriVax-JE): phase 2 clinical trials for safety and immunogenicity, effect of vaccine dose and schedule, and memory response to challenge with inactivated Japanese encephalitis antigen J Infect Dis 2003 14 8 1213 1230 10.1086/378356 14551893

[B6] Monath TP McCarthy K Bedford P Johnson CT Nichols R Yoksan S Marchesani R Knauber M Wells KH Arroyo J Guirakhoo F Clinical proof of principle for ChimeriVax: recombinant live, attenuated vaccines against flavivirus infections Vaccine 2002 14 7–8 1004 1018 11803060 10.1016/s0264-410x(01)00457-1

[B7] Torresi J McCarthy K Feroldi E Méric C Immunogenicity, safety and tolerability in adults of a new single-dose, live-attenuated vaccine against Japanese encephalitis: Randomised controlled phase 3 trials Vaccine 2010 14 50 7993 8000 10.1016/j.vaccine.2010.09.035 20934459

[B8] Chokephaibulkit K Sirivichayakul C Thisyakorn U Sabchareon A Pancharoen C Bouckenooghe A Gailhardou S Boaz M Feroldi E Safety and immunogenicity of a single administration of live-attenuated Japanese encephalitis vaccine in previously primed 2- to 5-year-olds and naive 12- to 24-month-olds: multicenter randomized controlled trial Pediatr Infect Dis J 2010 14 12 1111 1117 10.1097/INF.0b013e3181f68e9c 20856164

[B9] Chen WR Tesh RB Rico-Hesse R Genetic variation of Japanese encephalitis virus in nature J Gen Virol 1990 14 2915 2922 10.1099/0022-1317-71-12-2915 2273391

[B10] Chen WR Rico-Hesse R Tesh RB A new genotype of Japanese encephalitis virus from Indonesia Am J Trop Med Hyg 1992 14 61 69 1322071 10.4269/ajtmh.1992.47.61

[B11] Uchil PD Satchidanandam V Phylogenetic analysis of Japanese encephalitis virus: envelope gene based analysis reveals a fifth genotype, geographic clustering, and multiple introductions of the virus into the Indian subcontinent Am J Trop Med Hyg 2001 14 3 242 251 11561712 10.4269/ajtmh.2001.65.242

[B12] Oya A Japanese Encephalitis Vaccine Acta Paediatr Jpn 1988 14 175 184 10.1111/j.1442-200X.1988.tb02516.x 2854351

[B13] Hombach J Solomon T Kurane I Jacobson J Wood D Report on a WHO consultation on immunological endpoints for evaluation of new Japanese encephalitis vaccines, WHO, Geneva. 2–3 September 2004 Vaccine 2005 14 45 5205 5211 10.1016/j.vaccine.2005.07.002 16055233

[B14] Van Gessel Y Klade CS Putnak R Formica A Krasesub S Spruth M Cena B Tungtaeng A Gettayacamin M Dewasthaly S Correlation of protection against Japanese encephalitis virus and JE vaccine (IXIARO) induced neutralizing antibody titers Vaccine 2011 14 35 5925 5931 10.1016/j.vaccine.2011.06.062 21723353

[B15] Reisler RB Danner DK Gibbs PH Immunogenicity of an inactivated Japanese encephalitis vaccine (JE-VAX) in humans over 20 years at USAMRIID: using PRNT50 as an endpoint for immunogenicity Vaccine 2010 14 2436 2441 10.1016/j.vaccine.2009.12.080 20060946

[B16] Schuller E Jilma B Voicu V Golor G Kollaritsch H Kaltenbock A Klade C Tauber E Long-term immunogenicity of the new Vero cell-derived, inactivated Japanese encephalitis virus vaccine IC51 six and 12 month results of a multicenter follow-up phase 3 study Vaccine 2008 14 4382 4386 10.1016/j.vaccine.2008.05.081 18599165

[B17] Fulmali PV Sapkal GN Athawale S Gore MM Mishra AC Bondre VP Introduction of Japanese Encephalitis Virus Genotype I, India Emerg Infect Dis 2011 14 319 321 10.3201/eid1702.100815 21291622 PMC3204761

[B18] Ma SP Yoshida Y Makino Y Tadano M Ono T Ogawa M A major genotype of Japanese encephalitis virus currently circulating in Japan Am J Trop Med Hyg 2003 14 151 154 13677370

[B19] Zhang JS Zhao QM Guo XF Zuo SQ Cheng JX Jia N Wu C Dai PF Zhao JY Isolation and genetic characteristics of human genotype 1 Japanese encephalitis virus, China, 2009 PLoS One 2011 14 1 e16418 10.1371/journal.pone.0016418 21283590 PMC3026811

[B20] Erra EO Askling HH Yoksan S Rombo L Riutta J Vene S Lindquist L Vapalahti O Kantele A Cross-Protective Capacity of Japanese Encephalitis (JE) Vaccines Against Circulating Heterologous JE Virus Genotypes Clin Infect Dis 2013 14 267 270 10.1093/cid/cis883 23074319 PMC3526254

[B21] Fan YC Chen JM Chiu HC Chen YF Lin JW Shih CC Chen CM Chang CC Chang GJJ Chiou SS Partially neutralizing potency against emerging genotype I virus among children received formalin-inactivated Japanese encephalitis virus vaccine PLoS Negl Trop Dis 2012 14 9 e1834 10.1371/journal.pntd.0001834 23029592 PMC3459827

[B22] Feroldi E Pancharoen C Kosalaraksa P Watanaveeradej V Phirangkul K Capeding MR Boaz M Gailhardu S Bouckenooghe A Single-dose, live-attenuated Japanese encephalitis vaccine in children aged 12–18 months: randomized, controlled phase 3 immunogenicity and safety trial Hum Vaccin Immunother 2012 14 929 937 10.4161/hv.20071 22777096

[B23] Chambers TJ Nestorowicz A Mason PW Rice CM Yellow fever/Japanese encephalitis chimeric viruses: construction and biological properties J Virol 1999 14 3095 3101 10074160 10.1128/jvi.73.4.3095-3101.1999 PMC104070

[B24] WHO Expert Committee on Biological Standardization WHO Technical Report Series 910, Annex 3 Fifty-second report 2002 Geneva: World Health Organization 66 98 15497598

[B25] Jia L Wang Z Yu Y Protection of SA14-14-2 live attenuated Japanese encephalitis vaccine against the wild-type JE viruses Chin Med J (Engl) 2003 14 941 943 12877812

[B26] Mason PW Dalrymple JM Gentry MK McCown JM Hoke CH Burke DS Fournier MJ Mason TL Molecular characterization of a neutralizing domain of the Japanese encephalitis virus structural glycoprotein J Gen Virol 1989 14 2037 2049 10.1099/0022-1317-70-8-2037 2549181

[B27] Shimoda H Mahmoud HYAH Noguchi K Terada Y Takasaki T Shimojima M Maeda K Production and characterization of monoclonal antibodies to Japanese encephalitis virus J Vet Med Sci 2013 14 1077 1080 10.1292/jvms.12-0558 23519938

[B28] Kurane I Takasaki T Immunogenicity and protective efficacy of the current inactivated Japanese encephalitis vaccine against different Japanese encephalitis virus strains Vaccine 2000 14 33 35 10821971 10.1016/s0264-410x(00)00041-4

[B29] Chen YY Fan YC Tu WC Chang RY Shih CC Lu IH Chien MS Lee WC Chen TH Chang GJ Chiou SS Japanese encephalitis virus genotype replacement, Taiwan, 2009–2010 Emerg Infect Dis 2011 14 2354 2356 10.3201/eid1712.110914 22172307 PMC3311176

[B30] Pan XL Liu H Wang HY Fu SH Liu ZH Zhang HL Li MH Gao XY Wang JL Sun XH Lu XJ Zhai YG Meng WS He Y Wang HQ Han N Wei B Wu YG Feng Y Yang DJ Wang LH Tang Q Xia G Kurane I Rayner S Liang GD Emergence of genotype I of Japanese encephalitis virus as the dominant genotype in Asia J Virol 2011 14 9847 9853 10.1128/JVI.00825-11 21697481 PMC3196406

[B31] Sarkar A Banik BK Mukhopadhyay SK Chatterjee S Envelope protein gene based molecular characterization of Japanese encephalitis virus clinical isolates from West Bengal, India: a comparative approach with respect to SA14-14-2 live attenuated vaccine strain BMC Infect Dis 2013 14 368 381 10.1186/1471-2334-13-368 23927571 PMC3751164

